# Follow up in a developing country of patients with complete atrio-ventricular block

**DOI:** 10.5830/CVJA-2012-059

**Published:** 2012-11

**Authors:** JC Tantchou Tchoumi, Sara Foresti, Pierpaolo Lupo, Cappato Riccardo, Gianfranco Butera

**Affiliations:** Cardiac Centre, St Elizabeth Catholic General Hospital, Kumbo, Cameroon; Department of Electrophysiology, Policlinico San Donato IRCCS, Milan, Italy; Department of Electrophysiology, Policlinico San Donato IRCCS, Milan, Italy; Department of Electrophysiology, Policlinico San Donato IRCCS, Milan, Italy; Department of Pediatric Cardiology and Cardiac Surgery, Policlinico San Donato IRCCS, Milan, Italy

**Keywords:** complete atrio-ventricular block, follow up, cardiac centre

## Abstract

**Aim:**

The purpose of the study was to assess the incidence and survival rate of patients with complete atrio-ventricular block in the cardiac centre of St Elizabeth Catholic General Hospital, Kumbo, Cameroon.

**Methods:**

Between 2009 and 2011, 26 patients with complete atrio-ventricular block were diagnosed at our institution. Complete atrio-ventricular block was defined as complete heart block, diagnosed by echocardiographic or electrocardiographic documentation of the dissociation between electrical activity of the atria and ventricles. Hospital charts, electrocardiograms (ECG), echocardiography and chest radiography were reviewed.

**Results:**

The triad of symptoms that pointed to the diagnosis of complete atrio-ventricular block was mainly fatigue, shortness of breath on mild physical exertion, and dizziness. The median age at diagnosis was 65 ± 15 years. The escape rhythm showed a narrow QRS complex in 35.2% of patients, whereas wide QRS complexes were seen in 64.8%. In only 15 patients were pacemakers implanted: dual-chamber in 10 and single-chamber in five cases, depending on the availability of the pacemakers. During the observational period, five non-implanted patients died, giving a mortality rate of 45%. We recorded no deaths in patients with pacemakers.

**Conclusion:**

In developing countries, natural selection is observed in patients with complete atrio-ventricular block. Lack of infrastructure and early detection, and financial limitations are the main problems faced in the follow up of these patients. Re-organisation of the public health system, new programmes for the prevention of cardiovascular diseases, and government subsidisation are needed in our milieu.

## Abstract

Over the past years, cardiac pacing has become the standard mode of therapy for heart block and its complications. The increasing use of cardiac pacemakers (PM) has been encouraged by improved and simplified techniques of permanent pacing, by the development of more dependable electrodes and pulse generators, and by increasing clinical experience and follow-up data, indicating a favourable effect on the prognosis and improved cardiovascular performance.^1^

Across Europe in 2005, the number of new implants of pacemakers ranged from 121 to 1 134 per million, and for implantable cardiac defibrillators from 1.18 to 226 per million.^2^ In countries of sub-Saharan Africa, patients with complete atrioventricular block (CAVB) and other indications for pacing are sent home because of non-availability of facilities for pacemaker implantations, limited availability of pacemakers, and high cost of the implantation procedure. The aim of the study was to assess the incidence and survival in patients with CAVB during a period of 16 months at the cardiac centre of St Elizabeth Catholic General Hospital, Shisong, Cameroon.

## Methods

CAVB was defined as a complete heart block, diagnosed by echocardiographic or electrocardiographic documentation of the dissociation between electrical activity of the atria and ventricles. Hospital charts, electrocardiogram (ECG), echocardiography and chest radiography were reviewed. We analysed X-rays for cardiomegaly, which was defined as cardiothoracic ratio > 0.5.

Between 2009 and 2011, 26 patients with complete atrio-ventricular block were diagnosed at our institution. Structural heart diseases were diagnosed as follows: eight patients had hypertensive cardiomyopathy, seven had mild mitral valve regurgitation with degenerative aetiology, five had moderate mitral valve regurgitation with post-rheumatic aetiology associated with moderate tricupid valve regurgitation, one case had post-surgical complete atrio-ventricular block, one case had severe pulmonary artery valve stenosis, and the rest of the patients had no cardiac pathology.

Local anaesthesia was given in the left subclavicular area using 20 ml of lidocaine. The left subclavian vein was punctured and the guidewire was inserted for monocameral pacemakers. Two punctures were performed when we intended to implant a bicameral pacemaker. A pocket was created at the left subclavian area. Through the 9 and 7 french introducers we sent, respectively, the right atrial and the right ventricular leads in the case of a bicameral pacemaker. Through the 7 french introducer we sent the ventricular lead for a monocameral pacemaker. These introducers were observed by means of radiography.

The intra-operative parameters are reported in Table 1. These parameters were optimised three months after the implantation. The leads were anchored with silk 2.0 and the pacemaker was connected. Two layers of stitches were put in: the first, subcutaneous with vicryl 2.0 and the second, intradermic with vicryl 3.0. The post-surgical wound was covered with a plaster.

**Table 1.** Intra-Operative Parameters

	*Sensing (mV)*	*Threshold (V)*	*Impedance of the lead (Ohm)*
Atrial lead	3.5 ± 0.5	0.75 ± 0.5	768 ± 13
Ventricular lead	9 ± 0.5	0.5 ± 0.5	810 ± 9

Being a descriptive study, the data are presented as means and standard deviation.

## Results

Symptoms and signs that pointed to the diagnosis of CAVB are listed in Table 2. Median age at diagnosis was 65 ± 15 years. The escape rhythm showed a narrow QRS complex in 35.2% of patients, whereas a wide QRS complex was seen in 64.8%. In only 15 patients were pacemakers implanted: dual-chamber in 10 and single-chamber in five cases, depending on the payment capacity of patients. Complications observed after implantation were dislodgement of the lead in one patient, haematoma in two cases, and infection of the pocket in one case.

**Table 2.** Clinical Characteristics Of Patients

Total number of patients (*n*)	26
Age at diagnosis (years)	65 ± 15
Symptoms	
dizziness (*n*)	19
shortness of breath (*n*)	15
fatigue (*n*)	23
Adam Stokes attack (*n*)	6
palpitations (*n*)	3
No symptoms (*n*)	3
Co-morbidity	
hypertension (*n*)	16
degenerative arthritis (*n*)	20
diabetes mellitus	6
Referred cases (*n*)	2
Age at implantation (years)	70 ± 10
Type of block	
paroxystic (*n*)	6
permanent (*n*)	20
Causes of death	
Adam Stokes attack (*n*)	2
cardiovascular accident (*n*)	1
unknown (*n*)	2

During the observational period, five non-implanted patients in NYHA class III died, giving a mortality rate of 45%. The six remaining patients were in NYHA class II. All the implanted patients are alive and in a better clinical condition than the non-implanted patients (Table 3).

**Table 3.** Symptoms At Follow Up

	*Implanted patients*	*Non-implanted patients*
Shortness of breath (*n*)	2	6
Dizziness (*n*)	-	3
Adam Stokes attack (*n*)	-	3
Palpitations (*n*)	-	5
Fatigue (*n*)	-	5
Death	0	5
NYHA class II (*n*)	10	6
NYHA class I (*n*)	5	0

Chest pains in one patient were intercostal neuralgia, with no ischaemic aetiology. Importantly, before implantation, nine patients were in NYHA class III, and six in class II. After the implantation, 10 were in NYHA class II and five in class I.

## Discussion

Occasionally, adult patients do not have any symptoms of complete atrio-ventricular block and the diagnosis is made by detecting a slow heart rate at a routine examination. The incidence of CAVB seems to be higher in Lome than in the Shisong cardiac centre, being respectively, 1 and 2% (*p* < 0.02).3 We diagnosed few patients during the observational period, probably due to natural selection.

The mortality registered in non-implanted cases was 45%, low compare to the mortality in Togo, which was 59% (*p* < 0.05).^3^ In tertiary centres in sub-Saharan Africa, the main cause of death is lack of finances for the procedure. Patients with the pathology must pay before the device will be implanted, the dual-chamber pacemaker being more expensive than the single chamber, which is why in Africa in general more patients have single-chamber pacemakers.^4^

Besides eliminating the risk of sudden death, reasons for an early PM implantation in patients with CAVB are prevention of morbidity, left ventricular dilatation and dysfunction, and mitral regurgitation. Permanent pacemakers provide effective relief of symptoms and are life-saving in patients with symptomatic heart block.

Since pacemakers are only implanted by cardiologists or cardiothoracic surgeons in tertiary hospitals, the rates of pacemaker implantation provide a readily auditable measure of tertiary healthcare.^5^ In developed countries, patients with a history of complete heart block are almost absent because of the progress in medicine orientated to early detection and treatment of the condition, whereas in developing countries with the lack of finances, infrastructures and human resources, many cases are encountered.^6^

In this context in Africa, the re-use of pacemakers from charity organisations is a good solution; it can be carried out without increased risk to the patients, provided a proper routine for technical control and sterilisation is followed. Re-use means substantial savings, which could possibly make advanced pacemaker treatment available to all eligible patients irrespective of age. Death is not necessarily the end for heart devices.^7^,^8^ In our case, all the pacemakers we used were new.

We noted that some of our patients were asymptomatic with very wide QRS complexes, strengthening the hypothesis of natural selection. Electrophysiological and genetic studies are important to understand the mechanism of natural selection. We also found that patients with post-rheumatic heart disease were well represented in our study, causing us to suspect involvement of the conduction tissue in that pathology, as it is the case in patients with Lyme disease.^9^

The clinical state of implanted patients improved more than that of patients without pacemakers. In developing countries, cardio-stimulation should be made a department of all cardiac centres, since many lives are saved using pacemakers as therapy in post-surgical, paroxystic or permanent complete atrio-ventricular blocks.

## Conclusion

In undeveloped countries characterised by natural selection of patients with complete atrio-ventricular block, mortality is high. Lack of infrastructure, early detection and financial limitations are the main problems faced in the follow up of these patients. Re-organisation of the public health system, new programmes of prevention of cardiovascular diseases, and government subsidisation are needed in our milieu.

## References

[1] Ekpe EE, Aghaji MA, Edaigbini SA, Onwuta CN (2008). Cardiac pacemaker treatment of heart block in Enugu a 5-year review.. Niger J Med.

[2] Ector H, Vardas P (2007). Current use of pacemakers, implantable cardioverter defibrillators, and resynchronization devices: data from the registry of the European Heart Rhythm Association.. Eur Heart J.

[3] Yayehd K, Ganou K, Tchamdja T (2011). Management of high-grade atrioventricular block in Lome, Togo.. Med Trop.

[4] Thomas MO, Oke DA, Ogunleye EO (2007). Bradypacing: indications and management challenges in Nigeria.. Pacing Clin Electrophysiol.

[5] Millar RN (2001). Cardiac Arrhythmia Society of South Africa 1998 survey of cardiac pacing in South Africa. Report of the working group on registries of the cardiac arrhythmia society of South Africa (CASSA).. Afr Med J.

[6] Zion MM, Marchand PE, Obel IWP (1973). Long-term prognosis after cardiac pacing in atrioventricular block.. Br Heart J.

[7] Linde CL, Bocray A, Jonsson H (1998). Re-used pacemakers – as safe as new? A retrospective case–control study.. Eur Heart J.

[8] Mitka M (2007). Death not necessarily the end for heart devices.. J Am Med Assoc.

[9] Mohindra R, Pannu HS, Mohan B, Kumar N (2004). Syncope in rheumatic fever.. Indian Heart J.

